# Diffusion of OXA-48 carbapenemase among urinary isolates of *Klebsiella pneumoniae* in non-hospitalized elderly patients

**DOI:** 10.1186/s12866-022-02443-y

**Published:** 2022-01-19

**Authors:** Sandra Šuto, Branka Bedenić, Saša Likić, Sara Kibel, Maja Anušić, Vladimira Tičić, Gernot Zarfel, Andrea Grisold, Ivan Barišić, Jasmina Vraneš

**Affiliations:** 1 Department of Clinical Microbiology, Andrija Štampar Public Health Institute, Zagreb, Croatia; 2 Department of Microbiology, University of Zagreb School of Medicine, Kišpatićeva 12, Zagreb, Croatia; 3 Clinical Department for Clinical and Molecular Microbiology, University Hospital Center Zagreb, Zagreb, Croatia; 4 Department of Transfusiology, University Hospital Center Osijek, Osijek, Croatia; 5 Institute for Hygiene, Microbiology and Environmental Medicine, Medical University Graz, Graz, Austria; 6 Austrian Institute for Technology, Vienna, Austria

**Keywords:** *Klebsiella pneumoniae*, OXA-48, Resistance, Long-term care facility, Urinary tract infection

## Abstract

**Background:**

Recently, a dramatic increase of *Klebsiella pneumoniae* positive for OXA-48 β-lactamases was observed first in the hospital setting and later in the long-term care facilities (LTCFs) and community in the Zagreb County, particularly, in urinary isolates. The aim of the study was to analyse the epidemiology and the mechanisms of antibiotic resistance of OXA-48 carbapenemase producing *K. pneumoniae* strains isolated from urine of non-hospitalized elderly patients.

**Results:**

The isolates were classified into two groups: one originated from the LTCFs and the other from the community. Extended-spectrum β-lactamases (ESBLs) were detected by double disk-synergy (DDST) and combined disk tests in 55% of the isolates (51/92). The ESBL-positive isolates exhibited resistance to expanded-spectrum cephalosporins (ESC) and in majority of cases to gentamicin. LTCFs isolates showed a significantly lower rate of additional ESBLs and consequential resistance to ESC and a lower gentamicin resistance rate compared to the community isolates, similarly to hospital isolates in Zagreb, pointing out to the possible transmission from hospitals.ESBL production was associated with group 1 of CTX-M or SHV-12 β-lactamases. Ertapenem resistance was transferable from only 12 isolates. *bla*_OXA-48_ genes were carried by IncL plasmid in 42 isolates. In addition IncFII and IncFIB were identified in 18 and 2 isolates, respectively. Two new sequence types were reported: ST4870 and ST4781.

**Conclusions:**

This study showed eruptive and extensive diffusion of OXA-48 carbapenemase to LTCFs and community population in Zagreb County, particularly affecting patients with UTIs and urinary catheters. On the basis of susceptibility testing, β-lactamase production, conjugation experiments, MLST and plasmid characterization it can be concluded that there was horizontal gene transfer between unrelated isolates, responsible for epidemic spread of OXA-48 carbapenemase in the LTCFs and the community The rapid spread of OXA-48 producing *K. pneumoniae* points out to the shortcomings in the infection control measures.

## Background

The dynamic spread of resistance to carbapenems in Gram-negative bacteria has prompted researchers to analyse carbapenem-resistance mechanisms and to carry out epidemiological and surveillance studies. *Klebsiella pneumoniae* is an important hospital pathogen and causative agents of health care associated infections, including catheter associated urinary tract infections (CAUTIs). Carbapenem resistance in *K. pneumoniae* is mainly attributed to the acquisition of carbapenemases although other mechanisms such as porin loss or upregulation of efflux pumps may play a role. Carbapenemases involved in acquired resistance to carbapenems in *K. pneumoniae* belong to Ambler class A serin β-lactamases (KPC, GES, SME, IMI, NMC), class B metallo-β-lactamases (MBL) of the IMP, VIM or NDM family and OXA-48-like β-lactamases belonging to the class D [1, 2]. OXA-48 was reported for the first time in Turkey in 2004 [3] and is now endemic in North Africa, Middle East, and India [4] and some European countries such as Turkey, Spain, France, Belgium, the Czech Republic and Malta [2, 5]. It mediates resistance to penicillin and their combinations with inhibitors (clavulanic acid or tazobactam), but spares third and fourth generation cephalosporins. The resistance to expanded-spectrum cephalosporins (ESC) is present in the isolates coharbouring extended-spectrum or AmpC β-lactamases (ESBLs) [6, 7]. Carbapenemase encoding genes are usually carried by plasmids and some of them are located within the transposable elements which strongly facilitate their transmission between DNA replicons. The fast dissemination of the OXA-48 β-lactamase is mediated by insertion sequence IS*1999* embedded in transposon Tn*1999* [8]*.*

Croatia belongs to the countries with high prevalence of carbapenem-resistant *K. pneumoniae* typical for Southeast Europe and has a complex epidemiology concerning carbapenemases. Over the past eight years the carbapenemases in Croatia have diversified; initially the VIM-1 was the dominant type of carbapenemase in *K. pneumonia* [9], but was later outnumbered by KPC [10] and finally by alarming diffusion of OXA-48. The first OXA-48 producing *K. pneumoniae* were isolates in 2011 [11], but an alarming spread in the hospitals was reported in 2013–2014 [12, 13]. Gradual increase of OXA-48 carbapenemase among carbapenem resistant *K. pneumoniae* was reported in the outpatient setting (including LTCF) in Zagreb County with the rate of 93% in 2015 (326/349) and 2016 (385/411) and 95% in 2017 (820/859) and 2018 (1064/1121). The general rate of OXA-48 among carbapenem resistant *K. pneumoniae* in Croatia ranged from 91.5% in 2015 and 2016 to 94% in 2017 and 2018. The study carried out in north Lebanon found the prevalence of OXA-48 producing *Enterobacteriaceae* of 1.7% in rectal swabs of LTCFs residents [14]. Similar study carried out in Italy reported high prevalence of KPC β-lactamases (6.7%) in rectal swabs of the LTCFs residents [15], while OXA-48 was not found. None of the published studies analysed the presence of carbapenemases in clinically relevant specimens. Andrija Štampar Public Health Institute is in charge for microbiology analysis of specimens collected in Zagreb County, thus covering population of about 1.000.000 people including LTCFs in City of Zagreb. The majority of outpatient OXA-48 isolates originated from urine of elderly patients with urinary catheters. Carbapenem-resistant isolates were sent to Reference laboratory in the University Hospital for Infectious Diseases for carbapenemase identification.

All OXA-48 producing organisms from urine of the elderly, non-hospitalized patients were further sent to the University Hospital Center Zagreb to analyse the epidemiology and the mechanisms of antibiotic resistance. Furthermore, the resistance patterns and resistance gene content of the isolates obtained in these two health care niches were compared. Large-scale molecular surveys on carbapenemases in LTCFs are still rare in East Europe owing to many difficulties, particularly high cost. Previous investigation analysed colonization with multidrug resistant bacteria in LTCFs. However, there are only few bibliographical references on carbapenemases associated with infections in LTCFs.

## Results

### Patients’ characteristics

In total, 92 K*. pneumonia* isolates confirmed as OXA-48 isolates were collected from residents of 19 LTCFs, and from elderly outpatients in “Dr Andrija Štampar” Public Health Institute (56 and 36 isolates, respectively). The patient’s age range was 61 to 97 (median 80; x = 79.5; SD ± 8.7). There were 34 males and 58 females. All isolates were collected from urine of patients with significant bacteruria (> 10^5^ CFU/ml) and pyuria (> 5 white blood cells/high power field or positive leukocyte esterase). The dynamic analysis showed that the number of OXA-48 isolates in elderly patients raised from only seven in 2015 to 61 in 2018.

**Fig. 1:**
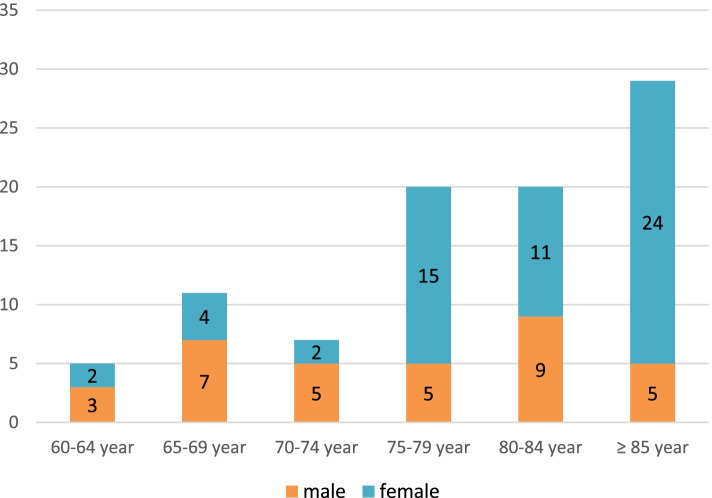
Distribution of patients according to age and gender

**Fig. 2:**
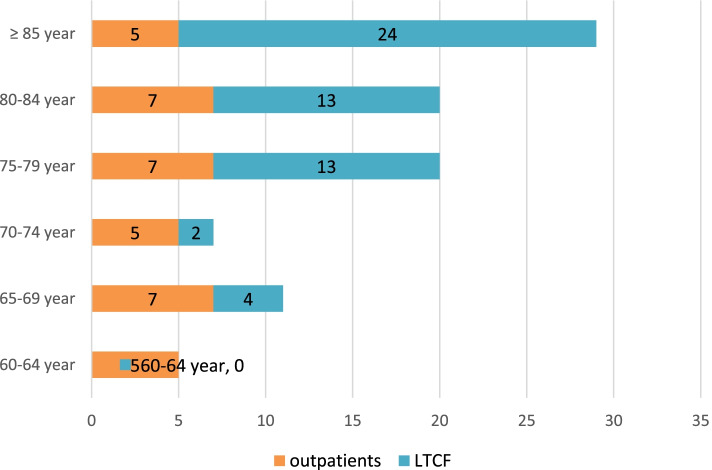
Distribution of patients according to the age and origin (LTCFs or outpatients)

**Fig. 3:**
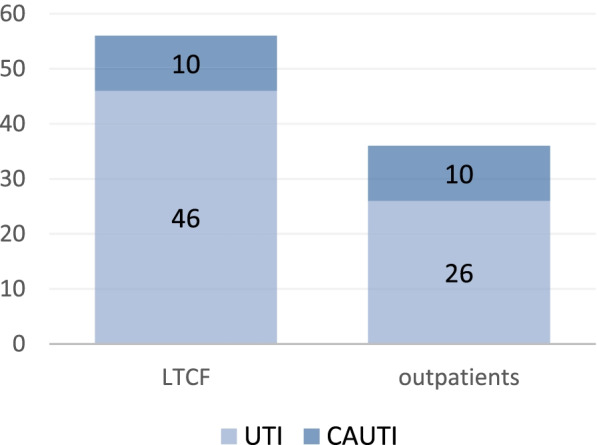
UTI vs CAUTI isolates in the LTCFs and outpatient setting

The proportion of female was significantly higher (63%; χ^2^ = 10.51; *p* < 0.01) (Fig. 1). Significantly higher proportion of institutionalized patients was recorded in the age ≥ 75 (χ^2^ = 15.58; *p* < 0.01) (Fig. 2). The proportion of CAUTI isolates in LTCFs was 18% vs 28% in outpatient isolates (Fig. 3). There was no clinically significant difference between distribution of UTIs and CAUTIs in the LTCFs and outpatient setting (χ^2^ = 1.27; *p* > 0.05). (Fig. 3).

### Antimicrobial susceptibility testing and phenotypic tests for detection of ESBLs, p-AmpC β-lactamases and carbapenemases

Extended-spectrum β-lactamases (ESBLs) were detected by double disk-synergy and combined disk test in 55% strains (51/92). Plasmid-mediated AmpC β-lactamases (p-AmpC) were suspected in 15 out of 92 isolates (16%) based on enlargement of the inhibition zones around ESC disks in the presence of PBA. Modified Hodge test and CIM test were positive in 84 (91%) and 90 (98%) isolates, respectively, indicating production of carbapenemase. Inhibitor-based tests with carbapenems and PBA was negative pointing out to the lack of KPC whereas EDTA produced significant enlargement of the inhibition zone around carbapenem disks (> 7 mm) in five isolates, being suspicious of MBL production.

The isolates were uniformly resistant to amoxicillin/clavulanic acid, piperacillin/tazobactam and ertapenem. High resistance rates were observed for ciprofloxacin (80/92–87%), cefazoline (75/92–82%) and cefuroxime (70/92–76%). Moderate resistance rates were recorded for ceftazidime (50/92–54%), cefotaxime (51/92–55%), ceftriaxone (51/92–55%), cefepime (44/92–48%) and gentamicin (34/92–37%). Imipenem and meropenem preserved good activity with only 18% (17/92) and 29% (27/92) resistant isolates, respectively. With EUCAST higher breakpoint of 8 mgl/L there were 5/92 (5.5%) and 7/92 (8%) resistant isolates to imipenem and meropenem, respectively. Moderate resistance rate of 58% (53/92) was observed for fosfomycin. If EUCAST criteria were applied with resistance breakpoint of > 32 the resistance rate would increase to 66% (70/92). Colistin resistance rate was relatively high with11% (10/92) resistant isolates with regard to the fact that it is the last line antibiotic for serious infections associated with carbapenemase producing organisms. Resistance to ESC was linked to ESBL production (*p* < 0.01). Comparison of resistance rates revealed significantly higher rates of additional ESBL and consequent resistance to ESC in outpatient than in LTCF isolates (78% vs 41%; χ^2^ = 11.95; *p* < 0.01). The rate of gentamicin and sulphamethoxazole/trimethoprim resistance was also markedly higher in community compared to LTCF isolates (47% vs 30% and 77% vs 64%, respectively) as shown in Tables 1 and 2.

**Table 1:** Characterization of OXA-48 carbapenemase—producing *K. pneumoniae* strains isolated from LCTF residents

No	**Strain number/** **protocol number**	Age / gender	CZ	CXM	CAZ	CTX	CRO	FEP	IMI	MEM	GM	CIP	FOS	COL	SXT	ESBL	MHT	CIM	ESBL	IS1999	P/ST
**1**	K2 22,694/2–17	86/F	> 128	> 128	> 128	> 128	> 128	32	2	4	8	> 128	16	0.5	R	+	+	+	SHV-12	+	L
**2**	K3 21,594/2–18	78/M	> 128	64	1	1	1	0.12	1	2	0.25	4	64	0.5	S	-	+	+	-	+	L,, FIB
**3**	K4 17,816/2–18	87/M	4	4	0.25	2	0.5	0.12	0.5	0.5	0.12	0.5	64	1	S	-	+	+	-	+	L
**4**	K8 58,646/2–18	80/F	4	4	0.5	0.25	0.5	0.12	1	1	0.25	4	32	0.5	S	-	+	+	-	+	L
**5**	K9 14,071/2–18	84/F	> 128	> 128	> 128	> 128	> 128	32	0.12	0.25	> 128	> 128	16	0.5	R	+	+	+	CTX-M gr. 1	+	FIIs
**6**	K12 32,372/2–18	85/F	> 128	> 128	1	0.25	0.5	0.12	0.5	1	0.12	> 128	128	0.5	R	-	+	+	-	+	L
**7**	K13 12,867/2–17	77/F	4	8	0.5	0.5	0.5	0.12	1	2	> 128	> 128	128	1	R	-	+	+	-	+	L
**8**	K14 35,500/2–18	78/F	> 128	> 128	16	> 128	> 128	16	2	4	> 128	2	128	0.5	R	+	+	+	SHV-12	+	L
**9**	K15 43,857/2–18	86/F	> 128	> 128	> 128	> 128	> 128	32	2	1	1	4	> 256	0.5	R	+	+	+	CTX-M gr. 1	+	L, FIIs
**10**	K17 52,663/2–18	82/F	> 128	> 128	0.5	1	1	2	2	8	8	> 128	128	0.5	R	-	+	+	-	-	
**11**	K18 31,720/2–18	86/F	> 128	> 128	32	> 128	> 128	16	0.5	2	0.25	> 128	256	0.25	R	+	+	+	SHV-12	-	FIIs
**12**	K19 27,078/2–18	83/F	> 128	> 128	0.5	0.25	0.25	0.5	2	0.25	0.5	4	32	0.25	S	-	+	+	-	-	
**13**	K20 48,278/2–18	95/F	> 128	> 128	64	> 128	> 128	32	0.5	0.5	> 128	> 128	> 256	0.25	R	+	+	+	SHV-12	-	
**14**	K21 55,024/2–18	92/F	2	8	0.5	0.25	0.25	0.5	0.25	0.25	8	4	> 256	0.25	S	-	+	+	-	-	
**15**	K22 47,812/2–18	89/F	> 128	> 128	64	> 128	> 128	32	0.25	0.25	32	> 128	256	0.12	R	+	+	+	CTX-M gr. 1	-	FIIs
**16**	K23 12,146/2–18	76/M	> 128	> 128	4	2	2	0.5	8	8	16	0.5	256	0.5	S	-	+	+	-	-	
**17**	K24 58,648/2–18	91/F	> 128	> 128	32	> 128	> 128	16	0.25	0.25	32	> 128	32	0.5	R	+	+	+	CTX-M gr. 1	-	L,FIIs
**18**	K25 39,335/2–17	86/F	> 128	> 128	16	> 128	> 128	8	2	0.25	16	> 128	256	0.25	R	+	+	+	CTX-M gr. 1	-	FIIs
**19**	K28 25,064/2–18	80/M	4	8	1	0.5	0.5	0.25	0.5	2	0.12	2	> 256	0.25	S	-	+	+	-	-	
**20**	K29 30,829/2–18	91/F	1	4	1	0.25	0.25	0.25	0.5	2	0.25	1	> 256	0.25	S	-	+	+	-	-	
21	K30 13,094/2–18	84/F	> 128	> 128	16	> 128	> 128	16	0.12	0.12	> 128	> 128	256	0.5	R	+	+	+	CTX-M gr. 1	+	FIIs
22	K33 58,558/2–16	74/M	2	4	0.5	1	1	0.5	2	0.5	0.06	16	256	0.5	R	-	+	+	-	-	ST37
23	K34 7317/2–18	86/F	> 128	> 128	> 128	4	32	32	8	4	0.06	16	> 256	0.12	R	+	+	+	CTX-M gr. 1	-	L, FIIs
24	K38 38,677/2–16	76/F	> 128	> 128	4	0.12	0.12	0.06	8	4	0.06	4	> 256	0.5	R	-	+	+	-	-	L
25	K42 2546/2–17	83/F	> 128	> 128	> 128	> 128	64	16	2	2	16	1	8	0.5	R	+	+	+	SHV-12	-	
26	K43 38,678/2–16	67/M	> 128	> 128	4	0.5	0.25	0.5	4	4	0.25	16	64	1	R	-	+	+	-	-	
27	K46 45,268/2–16	89/F	4	8	1	1	0.5	0.5	0.25	0.25	0.25	> 128	> 256	0.25	I	-	-	+	-	+	L
28	K51 38,674/2–16	97/F	> 128	> 128	4	0.5	1	0.25	2	1	0.06	> 128	> 256	0.25	R	-	+	+	-	+	ST629
29	K52 38,671/2–16	91/F	> 128	> 128	4	1	1	0.25	2	4	1	> 128	> 256	0.5	R	-	+	+	-	+	
30	K54 42,674/2–17	84/M	> 128	> 128	2	1	0.5	0.25	2	4	0.5	> 128	> 256	0.5	R	-	-	+	-	+	L/ ST1868
31	K57 56,675/2–17	91/M	> 128	> 128	32	64	32	16	2	2	32	> 128	> 256	0.12	R	+	+	+	SHV-12	+	L
32	K58 13,643/2–17	67/F	> 128	> 128	64	> 128	> 128	32	1	1	0.12	> 128	> 256	0.5	R	+	+	+	CTX-M gr. 1	+	L, ST431
33	K59 42,915/2–17	83/M	> 128	8	0.25	2	1	0.25	0.25	2	0.25	> 128	> 256	0.5	S	-	+	+	-	+	ST37
34	K60 39,501/2–17	87/F	> 128	> 128	0.25	2	0.5	0.12	2	1	0.25	> 128	> 256	0.25	R	-	+	+	-	+	L/ ST1868
35	K61 68,598/2–16	90/F	> 128	> 128	64	> 128	> 128	16	1	4	32	> 128	> 256	0.25	R	+	+	+	CTX-M gr. 1	+	L/ ST4871
36	K62 39,681/2–16	78/F	> 128	> 128	32	> 128	> 128	8	2	4	8	> 128	> 256	0.12	R	+	+	+	CTX-M gr. 1	+	L/ ST4871
37	K65 3690/2–16	90/F	> 128	> 128	1	2	2	1	8	4	0.06	> 128	128	0.5	R	-	+	-	-	+	
38	K66 41,954/2–17	71//M	> 128	4	0.5	0.5	1	0.25	2	0.5	0.06	4	16	0.5	R	-	+	-	-	+	
39	K67 44,848/2–17	76/F	> 128	64	> 128	> 128	> 128	64	2	1	16	64	256	0.5	S	+	+	+	CTX-M gr. 1	+	L
40	K68 7737/2–17	90/F	> 128	2	0.5	1	2	2	32	32	0.06	4	> 256	0.25	S	-	+	+	-	+	L
41	K70 39,928/2–17	79/M	2	4	1	1	0.5	0.25	2	0.5	0.06	4	128	0.12	S	-	+	+	-	+	L
42	K72 29,287/2–18	75/F	> 128	> 128	32	> 128	> 128	16	4	2	0.5	> 128	16	0.12	S	+	+	+	CTX-M gr. 1	+	L
43	K73 26,604/2–16	65/F	> 128	64	2	2	1	0.5	2	1	0.25	64	256	0.12	R	-	+	+	-	+	L
44	K74 153,634	84/F	> 128	> 128	64	> 128	> 128	64	0.5	0.5	8	> 128	256	0.06	R	+	+	+	CTX-M gr. 1	+	L
45	K76 33,291/2–15	82/F	4	4	1	1	0.5	0.25	4	1	0.5	2	32	0.5	S	-	+	+	-	+	
46	K78 46,243/2–17	91/F	2	8	0.5	1	1	1	0.5	0.5	0.5	2	256	0.25	S	-	-	+	-	+	L
47	K80 49,634/2–16	85/F	> 128	> 128	32	128	128	32	1	1	64	64	> 256	0.5	R	+	+	+	CTX-M gr. 1	+	
48	K82 51,572/2–17	67/F	> 128	> 128	2	2	1	2	16	16	0.5	> 128	64	1	S	-	+	+	-	+	
49	K85 25,219/2–18	91/F	> 128	> 128	0.25	2	2	1	2	1	0.5	16	32	1	S	-	+	+	-	+	
50	K86 52,010/2–17	77/M	2	8	0.25	0.5	2	0.5	4	1	0.5	4	64	1	S	-	+	+	-	+	
51	K87 56,464/2–18	81/M	2	4	0.5	0.5	1	0.06	2	1	0.5	4	> 256	1	S	-	+	+	-	+	
52	K96 31,658/2–18	80/F	2	4	0.5	1	2	0.5	1	0.5	1	4	> 256	8	R	-	+	+	-	-	
53	K98 68,595/2–16	77/F	> 128	> 128	128	128	128	32	1	4	32	> 128	> 256	16	R	+	+	+	CTX-M gr. 1	+	
54	K99 39,684/2-16a	76/F	> 128	> 128	128	128	128	32	1	4	32	> 128	256	8	R	+	+	+	CTX-M gr. 1	+	
55	K100 39,684/2-16b	76/F	1	4	0.5	1	2	1	4	4	32	> 128	> 256	8	R	-	+	+	-	+	
56	K102 79,539	89/F	> 128	> 128	8	32	32	8	4	8	0.5	> 128	64	0.5	R	+	+	+	CTX-M gr. 1	+	

*CZ* cefazoline, *CXM* cefuroxime, *CAZ* ceftazidime, *CTX* cefotaxime, *CRO* ceftriaxone, *FEP* cefepime, *IPM* imipenem, *MEM* meropenem, *GM* gentamicin, *CIP* iprofloxacin, *COL* colistin, *FOS* fosfomycin, *SXT* cotrimoxazole, *CIM* carbapenem inactivation method, *ESBL* combined disk test using cephalosporins alone and combined with clavulanate for detection of ESBLs, *MHT* modified Hodge test, *CIM* carbapenem-inactivation method, *P/ST* plasmid Inc group and sequence type

**Table 2:** Characterization of OXA-48 carbapenemase-producing *Klebsiella pneumoniae* strains from geriatric outpatients

No	**Strain number**	Age/ gender	CZ	CXM	CAZ	CTX	CRO	FEP	IMI	MEM	GM	CIP	COL	FOS	SXT	ESBL	MHT	CIM	ESBL	I*S1999*	P/ST
1	K1 4229/2–18	85/F	> 128	> 128	16	> 128	> 128	16	2	2	> 128	> 128	> 128	32	R	+	+	+	CTX-M gr. 1	+	L,FIIs
2	K5 12,944/2–18	75/M	> 128	> 128	64	> 128	> 128	16	1	2	0.25	0.5	0.5	32	S	+	+	+	CTX-M gr. 1	+	FIIs
3	K6 9057/2–18	87/M	> 128	> 128	> 128	> 128	> 128	64	0.5	1	2	2	0.5	64	S	+	+	+	CTX-M gr. 1	+	L,FIIs
4	K7 33,497/2–18	83/M	> 128	> 128	32	> 128	> 128	8	1	0.5	> 128	0.5	32	64	R	+	+	+	CTX-M gr. 1	+	L,FIIs
5	K10 25,085/2–18	84/F	> 128	> 128	64	> 128	> 128	16	0.12	2	0.5	> 128	0.5	> 256	R	+	+	+	CTX-M gr. 1	+	L,FIIs
6	K11 48,065/2–18	70/F	> 128	> 128	> 128	> 128	> 128	16	2	8	> 128	4	0.5	> 256	R	+	+	+	CTX-M gr. 1	-	FIIs
7	K16 58,160/2–18	81/M	> 128	> 128	64	> 128	> 128	16	1	1	0.5	> 128	0.5	16	R	+	+	+	CTX-M gr. 1	-	
8	K26 9938/2–18	87/M	> 128	> 128	32	> 128	> 128	16	0.25	2	2	> 128	0.5	256	R	+	+	+	CTX-M gr. 1	-	L,FIIs
9	K27 50,638/2–17	76/F	> 128	> 128	64	> 128	> 128	16	0.25	0.5	8	> 128	0.12	128	R	+	+	+	CTX-M gr. 1	-	L
10	K32 62,774/2–17	77/F	> 128	> 128	64	> 128	> 128	16	1	0.5	16	4	0.25	256	R	+	+	+	CTX-M gr. 1	-	L, Col, FIB, FII /ST29
11	K35 43,400/2–18	66/M	2	8	0.25	0.12	0.12	0.25	1	2	16	4	0.12	256	R	-	+	+	-	-	L
12	K36 6103/2–17	84/M	> 128	> 128	0.5	0.12	0.12	0.5	1	4	128	8	0.25	> 256	R	-	+	+	-	-	L
13	K39 51,869/2–16	91/M	> 128	> 128	> 128	> 128	64	8	1	2	0.06	4	0.5	> 256	R	+	+	+	SHV-12	-	L, FIIs
14	K44 66,350/2–16	72/M	> 128	> 128	0.5	0.25	0.25	0.5	4	4	0.25	8	0.25	256	R	-	-	+	-	-	
15	K48 37,545/2–16	77/F	> 128	> 128	> 128	> 128	64	16	2	2	0.5	> 128	0.5	> 256	R	+	+	+	CTX-M gr. 1	-	L
16	K49 5376/2–16	69/M	> 128	> 128	> 64	> 128	64	8	2	4	1	4	16	> 256	S	+	-	+	CTX-M gr. 1	-	
17	K50 27,771/2–17	73/M	> 128	> 128	1	2	0.5	0.5	0.25	2	0.06	> 128	0.5	> 256	S	-	-	+	-	-	
18	K53 45,871/2–16	75/F	> 128	> 128	64	> 128	> 128	32	4	8	16	> 128	0.5	> 256	R	+	+	+	CTX-M gr. 1	+	L/ST437
19	K55 57,452/2–17	83/M	> 128	> 128	0.25	2	0.5	0.12	2	2	0.25	> 128	0.25	> 256	S	-	-	+	-	+	L/ST727
20	K56 56,540/2–18	68/M	> 128	> 128	64	> 128	> 128	32	1	2	> 128	> 128	0.12	> 256	R	+	+	+	CTX-M gr. 1	+	L/ST4870
21	K64 37,429/2–16	68/F	> 128	> 128	> 128	> 128	> 128	> 128	2	1	1	16	0.12	128	S	+	+	+	CTX-M gr. 1	+	
22	K69 10,132/2–18	68/M	> 128	64	> 128	> 128	> 128	64	2	1	16	4	0.12	> 256	S	+	+	+	CTX-M gr. 1	+	L
23	K71 24,329/2–18	63/M	> 128	> 128	32	> 128	> 128	32	2	1	16	4	0.25	128	R	+	+	+	CTX-M gr. 1	+	
24	K75 40,425/2–15	74/F	> 128	> 128	> 128	> 128	> 128	> 128	4	8	32	2	0.25	64	R	+	+	+	CTX-M gr. 1	+	
25	K77 29,303/2–17	61/M	> 128	> 128	> 128	> 128	> 128	16	2	2	16	64	0.12	256	R	+	+	+	CTX-M gr. 1	-	
26	K79 56,293/2–17	78/F	> 128	> 128	16	64	64	32	0.5	0.06	0.5	64	0.5	32	R	+	+	+	CTX-M gr. 1	+	L
27	K83 44,694/2–18	64/F	> 128	> 128	> 128	> 128	> 128	> 128	1	0.5	0.5	> 128	1	> 256	R	+	+	+	CTX-M gr. 1	+	L, FIB, FII
28	K84 55,556/2–18	62/F	> 128	> 128	32	128	128	32	0.5	0.25	64	> 128	1	256	R	+	+	+	CTX-M gr. 1	+	L
29	K88 43,325/2–16	72/M	> 128	> 128	64	128	128	64	2	4	0.25	64	1	> 256	R	+	+	+	CTX-M gr. 1	+	
30	K90 40,020/2–16	84/M	16	16	1	2	2	0.5	2	2	16	32	0.5	64	R	-	-	+	-	-	
31	K94 28,126/2–18 a	65/M	> 128	> 128	32	128	128	16	1	0.25	64	4	4	32	R	+	+	+	CTX-M gr. 1	+	
32	K95 28,126/2–18 b	65/M	4	4	0.25	0.25	1	0.25	1	2	32	4	8	32	R	-	+	+	-	+	
33	K101 281,766	80/F	> 128	> 128	32	128	128	16	2	0.5	1	> 128	1	16	R	+	+	+	CTX-M gr. 1	+	
34	K103 20,886	75/F	> 128	> 128	128	128	128	8	4	4	2	> 128	0.5	32	R	+	+	+	CTX-M gr. 1	+	
35	K104 174,225	64/M	> 128	> 128	128	128	128	64	64	64	0.25	> 128	0.5	32	R	+	+	+	CTX-M gr. 1	+	
36	K105 9497	90/F	8	4	1	0,5	0,5	0,25	2	0,5	32	64	0,5	16	S	-	+	+	-	-	

*CZ* cefazoline, *CXM* cefuroxime, *CAZ* ceftazidime, *CTX* cefotaxime, *CRO* ceftriaxone, *FEP* cefepime, *IPM* imipenem, *MEM* meropenem, *GM* gentamicin, *CIP* iprofloxacin, *FOS* fosfmoycin, *COL* colistin, *SXT* cotrimoxazole, *ESBL* combined disk test using cephalosporins alone and combined with clavulanate for detection of ESBLs, *MHT* modified Hodge test, *CIM* carbapenem inactivation method, *P/ST* plasmid Inc group and sequence type

The isolates from LTCFs showed significantly higher MICs of carbapenems with resistance rates of imipenem and meropenem of 21% and 32%, being higher than those reported in community isolates (14% and 25%, *p* > 0.05). Surprisingly, the colistin resistance was higher among outpatients than in institutionalized patients (17% vs 7%; χ^2^ = 2.05, *p* > 0.05).

There was no statistically significant difference in the sensitivity of strains to gentamycin (χ^2^ = 0.05; *p* > 0.05), sulphamethoxazole/trimethoprim (χ^2^ = 1.88; *p* > 0.05), imipenem (χ^2^ = 0.83; *p* > 0.05), meropenem (χ^2^ = 0.54;*p* > 0.05) and colistin (χ^2^ = 2.05; *p* > 0.05) with respect to patients origin (LTCFs vs outpatients).

The percentage of MDR isolates was equal among LTCF residents and outpatients (34/56–61% vs 21/36–58%), while the percentage of XDR isolates was slightly higher among outpatients (15/56–27% in LTCFs residents vs 13/36–36% in outpatients).

Statistical significance in the distribution of MDR and XDR strains between LTCF residents and outpatients was not detected (χ^2^ = 0.52; *p* > 0.05).

### Conjugation

Eleven out of 51 ESBL isolates transferred cefotaxime resistances to *E. coli* recipient strain. The transconjugants exhibited similar resistance phenotype to β-lactam antibiotics as their respective donors. Sulphamethoxazole/trimethoprim resistance was co-transferred alongside with cefotaxime resistance from seven, gentamicin resistance from five and ciprofloxacin resistance from two strains. Twelve strains transferred ertapenem resistance to *E. coli* recipient strain with the frequency ranging from 2 × 10^–6^ to 1.5 × 10^–3^. Resistance determinants to non-β-lactam antibiotics were not co-transferred. The transformation experiments did not work out.

### Molecular characterization of resistance genes

Forty-four ESBL positive isolates (17 from LTCF and 27 from community) were found to possess genes for the group 1 of CTX-M-1 β-lactamases, and seven for SHV-12 (Tables 1 and 2). The same ESBL genes were also found in transconjugant strains obtained with cefotaxime as selective agent. *bla*_CTX-M_ genes were preceded by IS*Ecp* insertion sequence in 15 isolates. Comparison of LTCFs and community isolates revealed higher number of “old” ESBLs, SHV-12, among the institutionalized patients (6 vs 1). All isolates were positive for intrinsic *bla*_SHV_ genes. *bla*_OXA-48_ genes were the only carbapenem resistance determinant identified. Insertion sequence IS*1999* was found upstream of the *bla*_OXA-48_ genes in 59 isolates. Analysis of the flanking regions of *bla*_OXA-48_ gene revealed IS1R element between I*S1999* and the OXA-48 encoding gene. *qnr* genes were not found among fluoroquinolone resistant isolates. *mcr* genes were not detected in 10 colistin resistant isolates. Three cefoxitin resistant isolates were phenotypically positive for AmpC, but multiplex PCR for p-AmpC was negative. Similarly, five isolates positive in EDTA combined disk test tested negative in PCR for common MBLs in *K. pneumoniae*.

### Plasmid characterization

Forty-two isolates (27 from LTCF and 15 from outpatients) were shown to possess a plasmid which contained a replicon of the IncL group. The same plasmid was found also in the transconjugants obtained with ertapenem (Tables 1 and 2). The IncFIIs replicon was found in 19 isolates (11 from LTCF and 8 outpatients) whereas IncFIB was identified in two isolates. Three representative isolates were selected for whole genome sequencing and their plasmids analysed using the PlasmidFinder server (reference: https://doi.org/10.1128/AAC.02412-14). The identified plasmid contigs were then compared with publicly available plasmid sequences. All three isolates had an IncL plasmid with 100% sequence identity (SQI) to previously observed *bla*_OXA-48_-positive plasmids (average size ca. 63 kb) present in several pathogenic species such as *K. pneumoniae* (MK966142), *Klebsiella oxytoca* (CP064112), *Klebsiella aerogenes* (MN792918), *E. coli* (CP033880), and *Enterobacter hormaechei* (CP064115). *K. pneumoniae* 3 was found to possess six plasmids: a ColRNAI (100% SQI), an IncFIB(K)-like (99% SQI), an IncFIB(pKPHS1)-like (98% SQI), an IncFII(K)-like (98% SQI), an IncI2(Delta)-like (98% SQI), and an IncL (100% SQI) plasmid. The contig associated with the ColRNAI plasmid had 100% SQI with small plasmids (ca. 9 kb) mainly observed in *K. pneumoniae*. The sequences associated with the identified IncFIB(K)-like (100-200 kb; 99% SQI), IncFIB(pKPHS1)-like (109-112 kb; 99% SQI), and IncFII(K)-like plasmids (ca. 200 kb; 100% SQI) were previously reported in *K. pneumonia* strains. The sequences corresponding to the IncI2(Delta)-like plasmids were previously observed in *E. coli* strains (58 kb; 99% SQI). Five plasmids were identified in *K. pneumoniae* 32: a Col(pHAD28)-like (95% SQI), an IncFIB(K)-like (99% SQI), two IncFII(K)-like (96% and 98% SQI, respectively), and an IncL (100% SQI) plasmid. The sequences associated with the identified IncFIB(K)-like (ca. 20 kb; 100% SQI) and IncFII(K)-like plasmids (ca. 200 kb; 100% SQI) were previously reported in several *K. pneumonia* strains. The sequence of the Col(pHAD28)-like plasmid was previously not reported and showed lower similarities to several small plasmids (< 20 kb; < 97% SQI). *K. pneumoniae* 83 harboured three plasmids all with 100% SQI in the plasmid marker: IncFIB(K), IncFII and IncL. The sequence associated with the identified IncFIB(K) plasmid was previously reported in several plasmids and species of the *Klebsiella* genus (ca. 100-200 kb; 99%-100% SQI). The contig comprising the identified IncFII plasmid was 100% similar to plasmids (80-160 kb) dominantly identified in *E. coli* (15 hits), one *Citrobacter freundii* and one *K. pneumoniae* isolate.

### Multilocus- sequence- typing (MLST)

High diversity of STs was reported. Fourteen representative isolates were found to belong to 11 different STs (Tables 1 and 2). Two new STs were reported: ST4870 and ST4871. Three pairs of identical STs were recorded: ST 29, ST 37 and ST4871.

The links to the deposited STs are:

https://bigsdb.web.pasteur.fr/cgi-bin/bigsdb/bigsdb.pl?page=profileInfo&db=pubmlst_klebsiella_seqdef&scheme_id=1&profile_id=4870

https://bigsdb.web.pasteur.fr/cgi-bin/bigsdb/bigsdb.pl?page=profileInfo&db=pubmlst_klebsiella_seqdef&scheme_id=1&profile_id=4871

## Discussion

This study showed eruptive and extensive diffusion of OXA-48 carbapenemase to LTCFs and community population in Zagreb County, particularly affecting patients with UTIs and urinary catheters. Life expectancy in Croatia is rapidly increasing. Due to the ageing population, LTCFs which provide ongoing skilled nursing care to the residents and help meet both the medical and non-medical needs of the elderly individuals with chronic illness or disability became an important pillar in the Croatian health care system. Residents of the LTCFs often stay in the hospitals where they get colonized with MDR Gram-negative bacteria. Lapses in the infection control measures and wide use of carbapenems in geriatric population are responsible for the spread of carbapenemase-producing *Enterobacteriaceae* among the residents in the LTCF. Based on antibiotic susceptibility two groups were observed: one ESBL positive with high level resistance to majority of β-lactam antibiotics and also to gentamicin and ciprofloxacin and the other group without additional ESBL and susceptible to ESC, cefepime and gentamicin. P-AmpC β-lactamases were not found, similarly as in the previous report [13] although some of the isolates showed reduced susceptibility to cefoxitin, probably mediated by porin loss. The fact that a high proportion of the isolates carried additional ESBL genes and showed resistance to non-β-lactam antibiotics proves the capability of OXA-48 producing *K. pneumoniae* to accumulate other resistance genes. However, the majority of isolates, including ESBL producing organisms remained susceptible to imipenem, meropenem, amikacin and colistin in contrast to the recently published paper on OXA-48 producing isolates from blood cultures in Greece which all belonged to XDR or PDR phenotype [16]. Colistin resistance in LTCF has not yet reached the alarming rates observed in some hospitals in Croatia. *mcr* genes encoding plasmid-mediated colistin resistance were not found among colistin resistant isolates, indicating that resistance was probably due to mutations in *mgr*B genes, but the clarification of the resistance mechanism was beyond this study.

The isolates exhibited variable MICs of imipenem and meropenem ranging from 0.12 to 64 mg/L making the phenotypic detection difficult and unreliable. All isolates showed reduced susceptibility to ertapenem in disk-diffusion test which is used as screening method for carbapenemase production. The LTCFs isolates showed significantly lower proportion of ESBL positivity, but higher MICs of carbapenems compared to community isolates pointing out to the existence of two different groups; one from LTCFs and the other circulating in the community. Higher rate of ESBL positivity in outpatient setting was also linked to higher prevalence of aminoglycoside and sulphamethoxazole resistance carried on the same plasmid encoding ESBL. The LTCFs isolates were shown to be similar to the isolates from University Hospital Zagreb reported recently [13], with regard to being susceptible to ESC and free of ESBLs.

Due to the very variable level of carbapenem resistance microbiologists rely on the phenotypic tests. The majority of isolates exhibited resistance to ertapenem only, with MICs of imipenem and meropenem being in the susceptible range. For that reason, the laboratory detection of OXA-48 poses a serious challenge to clinical microbiologists. CIM test was shown to have higher sensitivity compared to Hodge test. Temocilin and faropenem disk and OXA-48 disk test are usually not done in Croatia, but bibliographical data show low specificity [17]. EDTA inhibitor-based test yielded positive results in five isolates, indicating production of an MBL. However, PCR for common MBLs in *K. pneumoniae* was negative. False positive phenotypic testing for MBLs was previously reported in *A. baumannii* [18, 19]. The explanation is that in the presence of EDTA oxacillinases are converted to less active state leading to an augmentation of the inhibition zone around the carbapenem disks. False positive phenotypic tests were also observed in PBA inhibitor-based test for p-AmpC.

High variability of carbapenem MICs could be attributable to variable expression of *bla*_OXA-48_ genes. Surprisingly, the MICs of meropenem were higher than of imipenem in the majority of isolates in spite of the fact that the imipenem is, generally, better hydrolysed by oxacillinases. The majority of the isolates were positive for IS*1999* upstream of the *bla*_OXA-48_ gene which is responsible for the mobilization of *bla*_OXA-48_ genes and enhances the expression of the gene. However, the rate of ertapenem resistance transferability was very low. Difficulties in transfer of plasmid from *K. pneumoniae* donor strains are likely due to unfavourable laboratory condition or defect in the transfer region. Because of this technical limitation, it was not possible to determine the location of *bla*_OXA-48_ genes in the majority of isolates. Analysis of the flanking regions of *bla*_OXA-48_ gene revealed similar structure as previously reported by Gianni et al. with IS1R element between I*S1999* and the OXA-48 encoding gene [8]. IS*Ecp1*-like element detected in approximately 1/3 of CTX-M positive isolates is a promoter for *bla*_CTX-M-15_ gene and is known to play a crucial role in its spread and mobility. However, in our study there was no correlation between the presence of an IS*Ecp1*-like element and the conjugation ability of the strain. Repeated attempts to transfer cefotaxime resistance were unsuccessful except in 15 ESBL positive isolates. The possible explanation is the chromosomal integration of *bla*_CTX.M-15_gene via ISE*cp1* insertion element as previously reported by Avgoulea [16].

The previous study performed on hospital OXA-48 K*. pneumoniae* isolates proved dissemination of OXA-48 due to the horizontal spread of plasmids carrying *bla*_OXA-48_ genes between unrelated isolates as a route of spread. In this study PFGE was not performed but the isolates were found to belong to different STs. Three pairs of identical STs were recorded: ST29, ST37 and ST4871. ST37 reported in this study was previously identified in KPC-2 K*. pneumoniae* in the early stage of dissemination in Croatia [20]. ST29 was previously associated with NDM-5 carrying hypervirulent strain of *K. pneumoniae* from China [21]. ST629 was previously identified in colistin resistant *K. pneumoniae* from Naples in Italy [22]. This pointed out to the fact that genetically related strains from different geographic areas can acquire different resistance traits under the selection pressure of antibiotics. Two new STs (4870 and 4871) were found in this study and deposited in the BIGSdb.

On the basis of susceptibility testing, β-lactamase production, conjugation experiments, MLST and plasmid characterization it can be concluded that there was horizontal gene transfer between unrelated isolates, responsible for epidemic spread of OXA-48 carbapenemase in the LTCFs and the community. IncL plasmids were found in the transconjugant strains and thus it can be concluded that they carry *bla*_OXA-48_ genes. Dissemination of *bla*_OXA-48_ genes due to horizontal spread of IncL plasmid was proved also in the Czech Republic [5].

This is in contrast with the previous studies performed on *P. mirabilis* and *A*. *baumannii* in LTCFs in which cross-transmission of related organisms under the selective pressure of antibiotics was proved.

Meropenem reduced susceptibility was transferable in the minority of isolates which is in contradiction with known efficient mobility of IncL plasmid.

The paper reports a detailed molecular survey on OXA-48 producing urinary *K. pneumoniae* isolates among geriatric population (institutionalized and non-institutionalized) in Zagreb County. The study addressed not only the problem of OXA-48 carbapenemase, but also the other enzymes compromising newer β-lactam antibiotics such as ESBLs. The results showed one of the highest rates of OXA-48 reported in Croatia and demonstrated how far the diffusion of OXA-48 can change the epidemiology of carbapenemases. The prevalence of carbapenemase producing *Enterobacteriaceae* in LTCFs depends on the geographical location, local epidemiology of antibiotic resistance, patient population, and the level of care provided. The majority of LTCFs in Croatia are still overcrowded precluding the optimal hygiene practices. The previous surveillance studies on carbapenemases in Croatia were carried out only in the hospital setting, but this study showed the need to include LTCFs in the future investigations. The development of new molecular methods has given the microbiologist the tools to detect origins and routes of spread of MDR bacteria in both hospital and institutional setting, as a step toward control and elimination of infection. Although the majority of isolates proved susceptible to imipenem and meropenem they should be administered with caution because of the possibility of developing mutants with high level carbapenemase production under the selection pressure of carbapenems. The study showed evolution of resistance in *K. pneumoniae* in LTCFs from ESBLs as the major resistance determinant to carbapenemases and colistin resistance. The insidious dissemination of OXA-48 producing *K. pneumoniae* in different components of health care system has major public health implications. The time will show whether it can mimic the global spread of CTX-M-15 that occurred in early 2000-ties. The dynamics analysis showed an increasing trend in the number of OXA-48 producing strains during the study period with alarming rates being achieved in the last two years. There are many gaps in our understanding of the development of antibiotic resistance in *K. pneumoniae* and its amazing capacity to accumulate resistance determinants. The species has special biological significance probably associated with specific antibiotic selection pressure, pathogenic fitness, gene mobility and other attributes.

## Conclusions

In conclusion, this study highlights the need to establish an antimicrobial resistance surveillance network for MDR *K. pneumoniae* in LTCFs to monitor the trends and new types of resistance mechanisms. Furthermore, the factors responsible for the selection and dissemination of L plasmids encoding OXA-48 need to be identified controlled and, when possible, prevented to avoid major outbreaks. Future efforts should be focused on screening activities, infection control measured tailored on the complex aspects of LTCFs and implementation of antibiotic stewardship programs.

## Material and methods

### Bacterial isolates

In total 92 K*. pneumoniae* isolates from urine (72), or catheter urine specimens (20) of elderly patients (> 61 years), in 19 LTCFs and outpatients in Zagreb County in the period from 2015 to 2018 were stored for research purpose and analysed. Ethical Permission for this study was issued by Ethical Committee of “Dr Andrija Stampar” Teaching Institute of Public Health (Classification number of permission is 641–01/20–01/01; registry number is 381–21-29).

The total number of OXA-48 producing *K. pneumoniae* in the study period was 2595 (1540 only OXA-48 and 1055 OXA-48 combined with ESBL). The isolates were identified to the species level by MALDI-TOF MS (matrix assisted laser desorption ionization-time of flight mass spectrometry) in the Andrija Štampar Public Health Institute. The production of an ESBL was detected by double disk synergy test (DDST) with disks containing ceftazidime (30 µg), cefotaxime (30 µg), ceftriaxone (30 µg), cefepime (30 µg) and amoxicillin/clavulanic acid (20/10 µg). All isolates that were confirmed as OXA-48 positive in the National Reference Centre were sent to University Hospital Centre Zagreb for further molecular analysis.

### Antimicrobial susceptibility testing and phenotypic tests for detection of ESBLs, plasmid-mediated AmpC β-lactamases (p-AmpC) and carbapenemases

The minimum-inhibitory concentrations (MIC) of various antibiotics were determined by the broth microdilution method in Mueller–Hinton broth and 98 well microtiter plates according to CLSI standard [23]. The following antibiotics were tested: amoxicillin alone and combined with clavulanate, piperacillin, piperacillin/tazobactam, cefazoline, expanded-spectrum cephalosporins or ESC (ceftazidime, cefotaxime, ceftriaxone), cefepime, imipenem, meropenem, ertapenem, gentamicin, ciprofloxacin and colistin. The susceptibility to fosfomycin was determined by agar dilution method. *Escherichia coli* ATCC 25,922 and *K. pneumoniae* 700,603 were used as quality control strains for MIC determination. The susceptibility to sulphamethoxazole/trimethoprim, tetracycline, and chloramphenicol of the transconjugants strains was determined by disk-diffusion test. The isolates were classified as multidrug-resistant (MDR), extensively drug-resistant (XDR) or pandrug-resistant (PDR) as described previously by Magiorakos et al. [24].

The production of ESBL was confirmed in DDST positive isolates by the CLSI combined disk test with disks containing ceftazidime, cefotaxime, ceftriaxone and cefepime with and without clavulanic acid [23]. Plasmid-mediated AmpC β-lactamases were detected by combined disk test using cephalosporin disks with 3-aminophenylboronic acid (PBA) [25]. A modified Hodge test (MHT) [26] and the carbapenem-inactivation method (CIM) [27] were used to screen for the presence of carbapenemases. Additionally, the isolates were tested by combined disk tests with imipenem and meropenem alone and combined with PBA, 0.1 M EDTA or both to screen for KPC, MBLs, or simultaneous production of KPC and MBL, respectively [28, 29].

### Conjugation and transformation

The transferability of ertapenem and cefotaxime resistance was determined by conjugation (broth mating method) employing *Escherichia coli* J65 recipient strain resistant to sodium-azide [30]. Equal volumes (1 ml) of donor and recipient cultures (10^9^ CFU/ml) were mixed in Brain–Heart infusion broth (5 ml) and incubated overnight at 35° C. The transconjugants were selected on MacConkey agar supplemented with either ertapenem (0.5 mg/L) or cefotaxime (2 mg/L) to inhibit the growth of the recipient strain and sodium azide (100 mg/L) to supress the growth of the donor cells. The frequency of conjugation was determined relatively to the number of donor cells. Co-transfer of resistance to gentamicin, tetracycline, sulfamethoxazole/trimethoprim, chloramphenicol, and ciprofloxacin was determined as well. The six isolates which did not yield transconjugants were subjected to transformation experiment as described previously [30]. Plasmids were extracted with Qiagen Mini Kit and transferred to electrocompetent *E. coli* A15R^−^ recipient strain. Transformants were selected on MacConkey medium containing 1 mg/L of ertapenem.

### Molecular detection of resistance genes

The genes encoding broad spectrum and extended-spectrum β-lactamases (*bla*_SHV_, *bla*_TEM_, *bla*_CTX-M_and *bla*_PER-1_) [31–34], plasmid-mediated AmpC β-lactamases [35], class A (*bla*_KPC_), class B carbapenemases (*bla*_VIM,_ *bla*_IMP_ and *bla*_NDM_), and class D (*bla*_OXA-48-like_) [36] and fluoroquinolone resistance genes (*qnr*A, *qnr*B, *qnr*S) [37] were amplified by PCR using primers and conditions, as described previously. The group of CTX-M β-lactamases was detected by multiplex PCR [38]. The presence of *mcr* genes encoding colistin resistance was detected by PCR as described previously [39]. For the sequencing purposes PCR products were purified using Qiaquick PCR purification kit (Qiagen, Hilden, Germany) and subjected to sequencing. Sequences were analysed according to the BLAST software. The positive control strains producing TEM-1, TEM-2 and SHV-1 and SHV-2 were kindly provided by Prof. Adolf Bauernfeind (Max von Pettenkofer Institute, Munich, Germany), CTX-M-15 by Prof. Neil Woodford (Health Protection Agency, London, UK) and OXA-48 by Dr. Yvonne Pfeifer (Robert Koch Institute, Wernigerode, Germany). PCR mapping was performed with primers for IS*1999* combined with forward and reverse primers for *bla*_OXA-48_ [8]. The size of the product was determined by gel electrophoresis in 1% agarose gel, (SeaKem LE agarose, Lonza, Rockland, USA) after staining with ethidium bromide. The amplicons of the three selected strains: 5376 (K49), 4229 (K1) and 39,684 (K99) were sequenced to determine the structures surrounding *bla*_OXA-48_ genes. Genetic context of *bla*_CTX-M_ genes was determined by PCR mapping with forward primer for IS*Ecp1* and IS*26* combined with primer MA-3 (reverse for *bla*_CTX-M_ genes) [40].

### Characterization of plasmids

Plasmids were extracted from donor strains and their respective transconjugants with Qiagen Miniprep Kit (Qiagen, Hilden Germany) according to the manufacturer’s instructions. After staining with ethidium bromide, the DNA was visualised by ultraviolet light. The size of the plasmid bands was determined by comparison with those of *E. coli* NTCC 50,192 yielding four bands of know sizes of 148, 64, 36 and 7 kb. PCR-based replicon typing (PBRT) [41] was applied to determine the plasmid content of the tested strains. Since it was observed previously that PBRT can be inefficient in identifying L/M plasmid incompatibility type, an updated method designated to identify and distinguish between IncL and IncM plasmids was applied [42]. Transconjugants obtained with cefotaxime were subjected to PCR for *bla*_ESBL_ genes while those obtained with ertapenem were tested for the presence of *bla*_CARB_ genes. Positive control strains for PBRT were kindly provided by Dr. A. Carattoli (Instituto Superiore di Sanita, Rome, Italy). Three isolates were selected for plasmid sequencing by whole genome sequencing (WGS): *K. pneumoniae* 3, *K. pneumoniae* 32 and *K. pneumoniae* 83.

### Multilocus sequence typing (MLST)

Multilocus sequence typing (MLST) was done in the Institute for Hygiene, Microbiology and Environmental Medicine of the Medical University in Graz, Austria.

Twelve isolates were genotyped by MLST according to Diancourt [43].

### Whole genome sequences (WGS)

WGS was applied to analyse the plasmid content of three representative strains: *K. pneumoniae* 3, *K. pneumoniae* 32 and *K. pneumoniae* 83. First, the strains were cultivated in Tryptic Soy Broth (TSB) and Casein-Peptone Soymeal-Peptone (CASO) Broth (Merck Millipore, MA, USA) at 37 °C overnight. Then, the genomic DNA was extracted using the QIAamp UCP Pathogen Mini Kit (Qiagen, Hilden Germany) according the manufacturer’s instructions. The DNA extracts were sent to the Next Generation Sequencing Facility of the Vienna Biocenter for sequencing using Illumina’s NextSeq1000 system according the manufacturer’s instructions. The single reads obtained were assembled and analysed using the webservers and services of the Center for Genomic Epidemiology (http://www.genomicepidemiology.org).

### Statistical analysis

Multiple comparisons between the groups were tested using χ^2^ test. A p-value of less than 0.05 was considered statistically significant.

## Data Availability

All data generated or analyzed during this study are included here and are available from the corresponding author on reasonable request. Public access to the University repository is open: http://medlib.mef.hr/ The links to the deposited STs on Pasteur MLST website are: (https://bigsdb.pasteur.fr/cgi-bin/bigsdb/bigsdb.pl?page=profileInfo&db=pubmlst_klebsiella_seqdef&scheme_id=1&profile_id=4870) and accession numbers (ST-4870 and ST-4871).

## Abbreviations

MIC: Minimum inhibitory concentration
MH: Mueller Hinton agar and broth
UTI: Urinary tract infection
CAUTI: Catheter associated urinary tract infections
CZ: Cefazoline
CXM: Cefuroxime
CAZ: Ceftazidime
CTX: Cefotaxime
CRO: Ceftriaxone
FEP: Cefepime
IPM: Imipenem
MEM: Meropenem
GM: Gentamicin
CIP: Iprofloxacin
COL: Colistin
FOS: Fosfomycin
SXT: Cotrimoxazole (sulphamethozaloe/trimethoprim)
CIM: Carbapenem inactivation method
ESBL: Combined disk test using cephalosporins alone and combined with clavulanate for detection of ESBLs
MHTMHT: Modified Hodge test
CIM: Carbapenem-inactivation method
P: Plasmid Inc group
ST: Sequence type
CAUTI: Catheter associated urinary tract infection
